# Opening the pathway towards a scalable electrochemical semi-hydrogenation of alkynols *via* earth-abundant metal chalcogenides†

**DOI:** 10.1039/d2sc04647d

**Published:** 2022-10-11

**Authors:** Kevinjeorjios Pellumbi, Leon Wickert, Julian T. Kleinhaus, Jonas Wolf, Allison Leonard, David Tetzlaff, Roman Goy, Jonathan A. Medlock, Kai junge Puring, Rui Cao, Daniel Siegmund, Ulf-Peter Apfel

**Affiliations:** Fraunhofer Institute for Environmental, Safety and Energy Technology UMSICHT Osterfelder Straße 3 D-46047 Oberhausen Germany ulf.apfel@umsicht.fraunhofer.de daniel.siegmund@umsicht.fraunhofer.de; Inorganic Chemistry I, Ruhr University Bochum Universitätsstraße 150 D-44780 Bochum Germany ulf.apfel@rub.de daniel.siegmund@rub.de; DSM Nutritional Products AG Wurmisweg 576 CH-4303 Kaiseraugst Switzerland; Key Laboratory of Applied Surface and Colloid Chemistry, Ministry of Education, School of Chemistry and Chemical Engineering, Shaanxi Normal University Xi'an 710119 China

## Abstract

Electrosynthetic methods are crucial for a future sustainable transformation of the chemical industry. Being an integral part of many synthetic pathways, the electrification of hydrogenation reactions gained increasing interest in recent years. However, for the large-scale industrial application of electrochemical hydrogenations, low-resistance zero-gap electrolysers operating at high current densities and high substrate concentrations, ideally applying noble-metal-free catalyst systems, are required. Because of their conductivity, stability, and stoichiometric flexibility, transition metal sulfides of the pentlandite group have been thoroughly investigated as promising electrocatalysts for electrochemical applications but were not investigated for electrochemical hydrogenations of organic materials. An initial screening of a series of first row transition metal pentlandites revealed promising activity for the electrochemical hydrogenation of alkynols in water. The most active catalyst within the series was then incorporated into a zero-gap electrolyser enabling the hydrogenation of alkynols at current densities of up to 240 mA cm^−2^, Faraday efficiencies of up to 75%, and an alkene selectivity of up to 90%. In this scalable setup we demonstrate high stability of catalyst and electrode for at least 100 h. Altogether, we illustrate the successful integration of a sustainable catalyst into a scalable zero-gap electrolyser establishing electrosynthetic methods in an application-oriented manner.

## Abstract 2

Holistic pathway for the sustainable electrochemical hydrogenation of alkynols – from robust metal sulphides to scalable zero-gap electrolysers.

## Graphical abstract

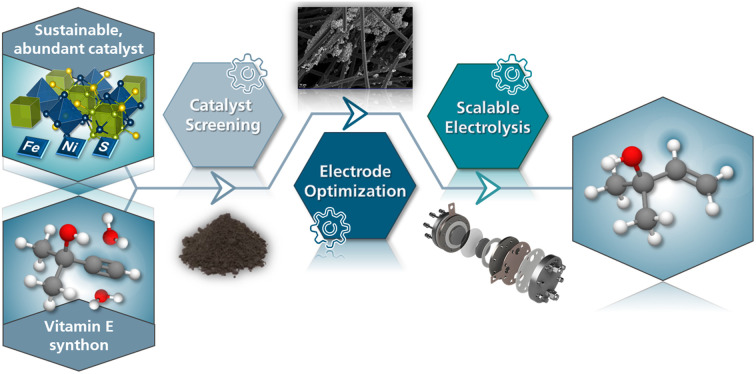

## Introduction

Hydrogenation reactions constitute some of the most important transformations in the chemical industry providing modern society with important synthons for the production of pharmaceuticals, fragrances, vitamins and margarines amongst others.^1,2^ Despite their large-scale industrial application, the use of molecular hydrogen under elevated pressures and temperatures, as well as the employment of expensive and scarce noble metal catalysts such as Pt or Pd, have made the development of greener alternatives increasingly more important in recent years.^3^

Recently, synthetic pathways *via* electrochemical means are gaining increasing attention as tuneable and sustainable replacement of thermal processes.^4^ In addition to their modular and scalable character, electrochemical approaches operate under mild conditions with high atom economy compared to their thermocatalytic counterparts.^5^ Regarding hydrogenation reactions in particular, electrochemistry can effectively mitigate safety hazards, transport and storage costs as well as carbon dioxide emissions associated with the supply of highly pressurized dihydrogen.^6,7^ In the case of electrosynthesis instead of gaseous H_2_, the solvent, ideally water, acts as the proton source, while the possibility to directly use electrons from renewables enables a sustainable implementation.^8^ By carefully tuning the applied potential, temperature and electrolyte, a variety of functional groups such as ketones,^9^ aldehydes, esters,^10^ olefins,^7^ alkynes,^11^ nitriles^12^ and oximes^13^ have been electrochemically hydrogenated in recent years.

Albeit their promising perspective, electrolysers for the electrochemical hydrogenation (ECH) often still use expensive noble metals such as Pd and Pt, deeming the discovery and implementation of novel and inexpensive alternatives crucial to accelerate the large-scale implementation of this technology.^14^ Along this line, transition metal chalcogenide-based electrocatalysts have recently gained interest as cheap and stable alternatives to noble metal electrocatalysts in numerous applications.^15^ In case of ECH reactions, a few monometallic sulphur-depleted transition metal chalcogenides such as CuS_*x*_ and CoS_2−*x*_ and Co_3_S_4−*x*_, have already shown elevated activity for the ECH of alkynes,^16^ unsaturated aldehydes^17^ and nitroarenes,^18^ respectively. Another class of transition metal chalcogenides that could show great promise for the field of ECH in terms of both catalytic activity as well as facile and rapid scalability are pentlandite (Pn) materials. Our group has shown that depending on the applied electrochemical conditions pentlandite-based electrocatalysts, M_9_X_8_ (M: Fe, Co, Ni; X: S, Se), possess the ability to generate reactive hydride species on the catalytic surface either leading to the generation of hydrogen or promoting the hydrogenation of acetonitrile to ethane.^19,20^ Regarding scalability, the synthesis of pentlandite materials has also been established by green and scalable mechanochemical methods^21^ revealing further possible perspectives towards an industrial implementation. Most significantly, a crucial advantage of the pentlandite structure over other transition metal chalcogenides is the ability to fine-tune structural and electronic properties of the employed catalysts *via* a plethora of metal (Fe/Co/Ni) and chalcogenide (S/Se) variations.^22–25^ Consequently, utilising this stoichiometric flexibility more active pentlandite catalysts for the hydrogen evolution reaction (HER), CO_2_ reduction and oxygen evolution (OER) reaction could be developed in recent years.^21–23,25,26^ Overall, this interesting subgroup of multi-functional electrocatalysts demonstrates a combination of ideal properties towards the application in the field of ECH.

**Fig. 1:**
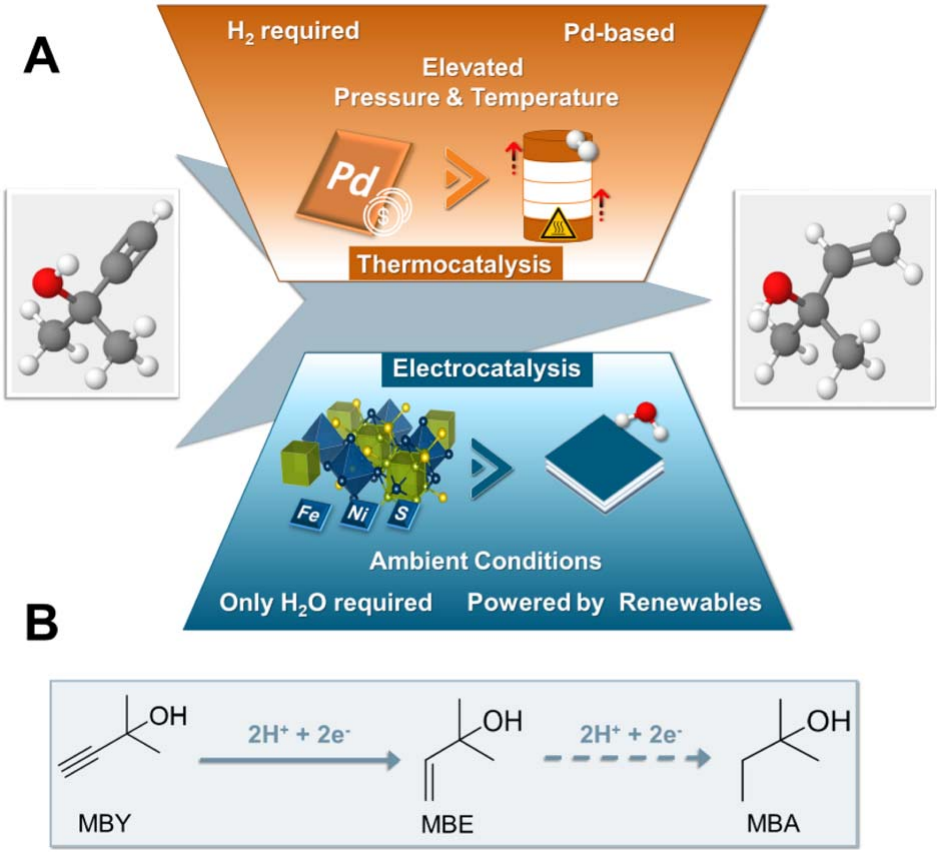
Schematic representation comparing the current state-of-the-art synthesis of MBY to the herein presented work based on pentlandite electrocatalyst (A). Equation depicting the ongoing hydrogenation processes within the ECH electrolyser (B).

Regarding conceivable substrates, we turned our focus to the substrate class of alkynols, globally playing a major role in the fields of vitamins and perfumes.^1^ More specifically, in order to investigate the capability of pentlandite catalysts towards ECH, we selected 2-methyl-3-butyn-2-ol (MBY) as a model substrate, which represents the main industrial synthon for vitamin A and E synthesis, reaching annual productions in the scale of thousands of metric tons.^1,27^ Currently, the industrial hydrogenation of MBY to the highly desired alkene, 2-methyl-3-buten-2-ol (MBE), is mainly performed in batch-reactors under elevated temperatures and pressures suffering from issues of sustainability. Specifically, the lack of green reductants, employment of Pd-based and Pb-containing catalysts, as well as the necessity for additional purification steps, *e.g.* separation of products from the catalysts, remain key drawbacks of this approach.^1^ Similarly, despite the ton-scale production of MBE, the minimization of unwanted over-hydrogenation to 2-methyl-3-buten-2-ol (MBA) or possible side reactions, such as dimerization among others, remain major bottlenecks and key topics of current research of MBY hydrogenation.^28^ Furthermore, whilst there have been some reports of a continuous MBY-hydrogenation there is still the need for further improvements.^29^ Most notably, despite its industrial importance attempts towards the generation of MBE by the more sustainable electrochemical route remain limited within academic or patent literature, with base metal catalysts being non-existent (Fig. 1).^30^

Herein, we report for the first time the electrochemical conversion of MBY (2-methyl-3-buty-2-ol) to the respective alkene MBE by a PGM-free catalyst in aqueous solution, without the supply of H_2_. The conversion of this important vitamin synthon is achieved through a series of robust and cheap transition metal chalcogenides based on the pentlandite structure. Going beyond screening experiments, we integrated the most active composition, Fe_3_Ni_6_S_8_, into industrially relevant zero-gap electrolysers, reaching yields of 70% for MBE at 240 mA cm^−2^ at a cell voltage of 3.0 V, being on par with current noble metal-based electrolysers, while providing a highly sustainable alternative to the noble-metal-based thermocatalytic state-of-the art.

## Results and discussion

### Screening of Fe_9−*x*_Ni_*x*_S_8_ –pentlandites for the ECH

Due to our previously gained experiences with this material class, we first focused on already reported materials with variable Ni/Fe ratios (Fe_9−*x*_Ni_*x*_S_8_). This class of catalysts allows us to vary the metal centres without leading to any significant structural changes of the crystal lattice.^22^ Following reported synthetic procedures for pentlandite materials, we prepared a series of Fe_9−*x*_Ni_*x*_S_8_ (*x* = 3–6, herein denoted as Ni_*x*_) catalysts, with different Ni/Fe ratios, *via* mechanochemical methods (Fig. S1†).^21^ Subsequently, aiming to investigate the intrinsic activity of the (Fe,Ni)_9_S_8_ catalysts, the obtained powders were pressed into pellets and mounted into electrodes with the help of a conductive non-catalytically active carbon glue. The polished pellet electrodes (*Ø* 3 mm, *A* = 0.071 cm^2^) were used as a working electrode in a three-electrode H-type PEEK cell, possessing a compartment volume of 15 mL (Fig. S2†). To directly probe the activity of our pure pentlandite materials while also approaching preparative relevant conditions, electrolytic experiments were performed at 100 mA cm^−2^ for 2 h. To guarantee sufficient conductivity during testing, our catholyte comprised of 0.3 M KOH and 1 M MBY in water. Notably, the employed reactant as well as the conceivable products are stable under these conditions. All obtained solutions were analysed *via* headspace GC-MS.

**Fig. 2:**
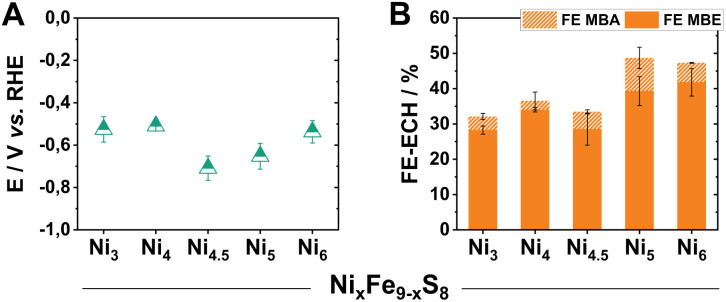
Half-cell potentials of the herein investigated Ni_*x*_ pellet electrodes after 2 h of electrolysis at 100 mA cm^−2^ (A). The respective faradaic efficiency values *via* GC-MS quantification (B).

Within the investigated series, all tested Pn-materials show good catalytic activity towards ECH in the employed aqueous electrolyte starting from Ni_3_, possessing a faradaic efficiency for the generation of MBE (FE_MBE_) of 28% accompanied by a FE_MBA_ of 10% (Fig. 2B). Transitioning to higher Ni-equivalents, the selectivity towards the ECH increases with Ni_5_ and Ni_6_ showing similarly elevated FE_MBE_ values of 40% at 100 mA cm^−2^, with the remaining percentages corresponding to H_2_ generation as evidenced by gas chromatography. Regarding the observed half-cell potentials, except for Ni_4.5_ and Ni_5_, all of the tested materials demonstrate low half-cell potentials of *ca.* −0.5 V *vs.* RHE at 100 mA cm^−2^ (Fig. 2A).

Inspired by these promising results, we extended our screening and elucidated the role of different metal atoms on the ECH in pentlandites. We subsequently also compared the activity of Ni_6_ with its cobalt analogues, Co_9_S_8_ (Co_9_), FeCo_8_S_8_ (FeCo_8_), and NiCo_8_S_8_ (NiCo_8_), which have been recently synthesized by our group as potential HER-catalysts.^31^ Interestingly, transitioning from a Fe/Ni-based system to a Co-based one has a major effect on the observed ECH selectivity. Specifically, Co_9_ shows the lowest activity towards ECH at a FE_MBE_ value of 19%, while the addition of either one equivalent of Ni or Fe leads to an enhancement of the observed ECH activity (Fig. S3†). Specifically, FeCo_8_ enables the production of MBE with a FE of 50%, while NiCo_8_ generates a mixture of hydrogenated products consisting of MBE (FE_MBE_ 25%) and MBA (FE_MBA_ 18%). Compared to their Ni/Fe-counterparts, Co-based variants also demonstrate a higher half-cell potential with FeCo_8,_ showing the largest value at −2.7 V *vs.* RHE. These clear differences not only within the MCo_8_S_8_ series but also the different Ni/Fe ratios demonstrate, that small alterations in the composition of our multi-metallic transition metal-rich chalcogenides offer the potential to control the catalytic selectivity and activity for the ECH.

Lastly, to provide a holistic picture within our screening investigation, we compared the activity of our materials to the performance of monometallic sulphides such as FeS and NiS (Fig. S4†). Although these analogues showed some activity for the conversion of MBY, with NiS showing a FE_MBE_ of 62%, they generally suffered from severe instability under the employed electrocatalytic conditions, a behaviour already observed in reports for the hydrogen evolution reaction.^20^ Consequently, the monometallic sulphides were omitted for this investigation.

Since Ni_6_ showed the highest selectivity for the ECH, an elevated MBE : MBA ratio as well as a low half-cell potential within our initial screening (Table S1†), we selected it as our catalyst of choice in further comparisons as well as our following implementation into scalable electrolysers.

### Implementation of pentlandite materials in zero-gap electrolysers

Having successfully demonstrated that pentlandite-materials efficiently hydrogenate MBY, we shifted our focus towards achieving a link between catalyst development and implementation in scalable cell designs. In recent years, zero-gap electrolysers, have shown great promise in electrolytic applications not only for the H_2_ generation or the CO_2_ electroreduction, but also for the ECH.^32^ Here, an ion-exchange membrane is pressed in between the porous anode and cathode electrodes. This configuration decreases ohmic resistances, allows for a better control of the reaction environment, facilitates reactant transport, and, more importantly, permits the continuous large-scale operation of such organic transformations.^33^

**Fig. 3:**
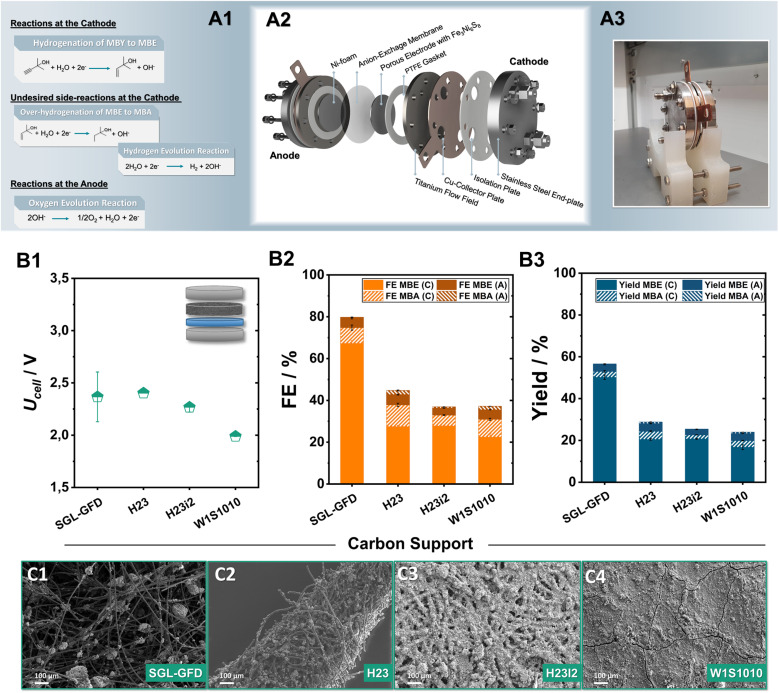
Reaction schemes of the different half-cell reactions during electrolysis (A1). Schematic representation (A2) and picture of the 12.57 cm^2^ electrolyzer employed in this work (A3). Investigation of the role of the employed carbon-support for the semi-hydrogenation of MBY at 80 mA cm^−2^ for 2 h of electrolysis. The obtained cell voltage (B1), faradaic efficiency (B2) and yield (B3) through ^1^H-NMR quantification. Here, FE_MBX_ (C) and FE_MBX_ (A) (X: E, A) correspond to the FE values of ECH products detected in the catholyte and anolyte after electrolysis, respectively. Scanning electron microscopy (SEM) analysis of the resulting catalytic layer on SGL-GFD (C1), H23 (C2), H23i2 (C3) and W1S10101 (C4) at a magnification of 100×.

Focusing on the minimization of noble metals, our investigation mainly employs anion-exchange membranes, allowing for the employment of robust and cost-effective anodes such as Ni-foam for O_2_-formation. The herein investigated membrane electrode assemblies (MEAs) were implemented into an in-house built zero-gap electrolyser, with an active area of 12.57 cm^2^, consisting of an anion-exchange membrane sandwiched between a Ni-foam anode and carbon supports coated with sulfidic catalysts on the cathode, respectively (Fig. 3A1–A3 and S5†). During electrolysis, 1 M MBY:0.3 M KOH in H_2_O as well as 2 M KOH were used as catholyte and anolyte respectively, with the electrolyte being continuously recirculated while a current density of 80 mA cm^−2^ was applied for 2 h. Furthermore, we monitored the substrate and product crossover through the AEM by NMR analysis of both electrolyte compartments (Fig. S6–S9†). The herein discussed results will focus mainly on faradaic efficiencies obtained from the catholyte. The concentration of ECH-products in the anolyte reservoir as well as the respective FE value will only be mentioned when significant crossover was observed.

A key point in our optimization of the ECH in zero-gap electrolysers is the carbon porous transports layer (PTL) employed in the cathode. Although the role of the PTL has been rigorously investigated in the fields of fuel cells as well as electrolysers for the CO_2_ reduction or hydrogen generation,^34^ in the case of ECH reactions, it currently constitutes an unexplored parameter. To address this issue and optimize the transport of organic substrates to and of the generated products from the active centres, Ni_6_ was airbrushed onto a series of commercially available carbon supports; W1S1010, H23, H23i2, and Sigracell GFD 2.5, employing 10 wt% PTFE as a binder. A detailed list of the different PTL properties and vendors can be found in the ESI (Table S2†).

Comparing the two carbon paper substrates H23 and H23i2, it is evident that the hydrophobic treatment plays a minimal role in steering the observed ECH-activity (Fig. 3B2). Both, H23 and its hydrophobically treated counterpart H23i2 demonstrate similar FE_MBE_ values, close to 28% alongside the generation of 4% of MBA. The additional implementation of a microporous layer (MPL) accompanied by a hydrophobic treatment in the case of W1S1010 has a negligible effect on the obtained FE_MBE_. Notably, transitioning from traditionally employed carbon paper and cloth materials to a porous carbon felt used in redox-flow batteries leads to a significant enhancement of the observed ECH activity.^35^ Applying a current density of 80 mA cm^−2^, the use of the Sigracell GFD carbon felt (SGL-GFD) as a conductive support, enables the highest activity for the ECH, reaching FE_MBE_ values of 67% alongside a FE_MBA_ of 8% at a full cell voltage of only 2.4 V, being on par with or outcompeting noble-metal containing MEAs for the ECH.^36^ The corresponding yields after 2 h of electrolysis were determined to be 53% for MBE and 3% for MBA in the catholyte, confirming appreciable selectivity for alkene formation with a rate of 0.12 mL_MBE_ h^−1^ cm^−2^ at 80 mA cm^−2^ (Fig. 3B3). Regarding crossover of organic products through the membrane by means of either electro-osmosis, or membrane swelling, in the case of all Ni_6_-based carbon electrodes 5% of the total FE_MBE_ and 2% of the total FE_MBA_ quantified were detected in the anolyte reservoir after 2 h of electrolysis. Moreover, analysis of the observed gaseous products *via* online GC-MS for the SGL-GFD shows that the remaining FE percentages can be attributed to hydrogen evolution (Fig. S9†).

Analysis of the generated electrodes *via* SEM-EDX reveals that the different carbon supports result in significantly different morphologies of the catalytic layer (Fig. 3C1–C4). As observed by SEM/EDX-measurements (Fig. S10–S15†), W1S1010 carbon cloth, which shows the lowest FE for the ECH in tested series, shows the most compact catalytic layer, possibly due to the existence of a microporous layer (MPL) on the gas diffusion layer (GDL). Moving towards H23 and H23i2 the Ni_6_ particles appear to be spread through the surface of the carbon material in the form of spherical agglomerates. Similarly, in case of the SGL-felt, the air-brushed particles are not only located on the upper surface but are also embedded within the 3D structure of the felt forming catalytic clusters on the intersections of the carbon fibres. The formation of these dispersed Ni_6_-PTFE clusters alongside the significantly larger pores of the SGL-GFD could be responsible for the almost doubled activity through better transport of organic reactants and products to and from the catalytic centres.

**Fig. 4:**
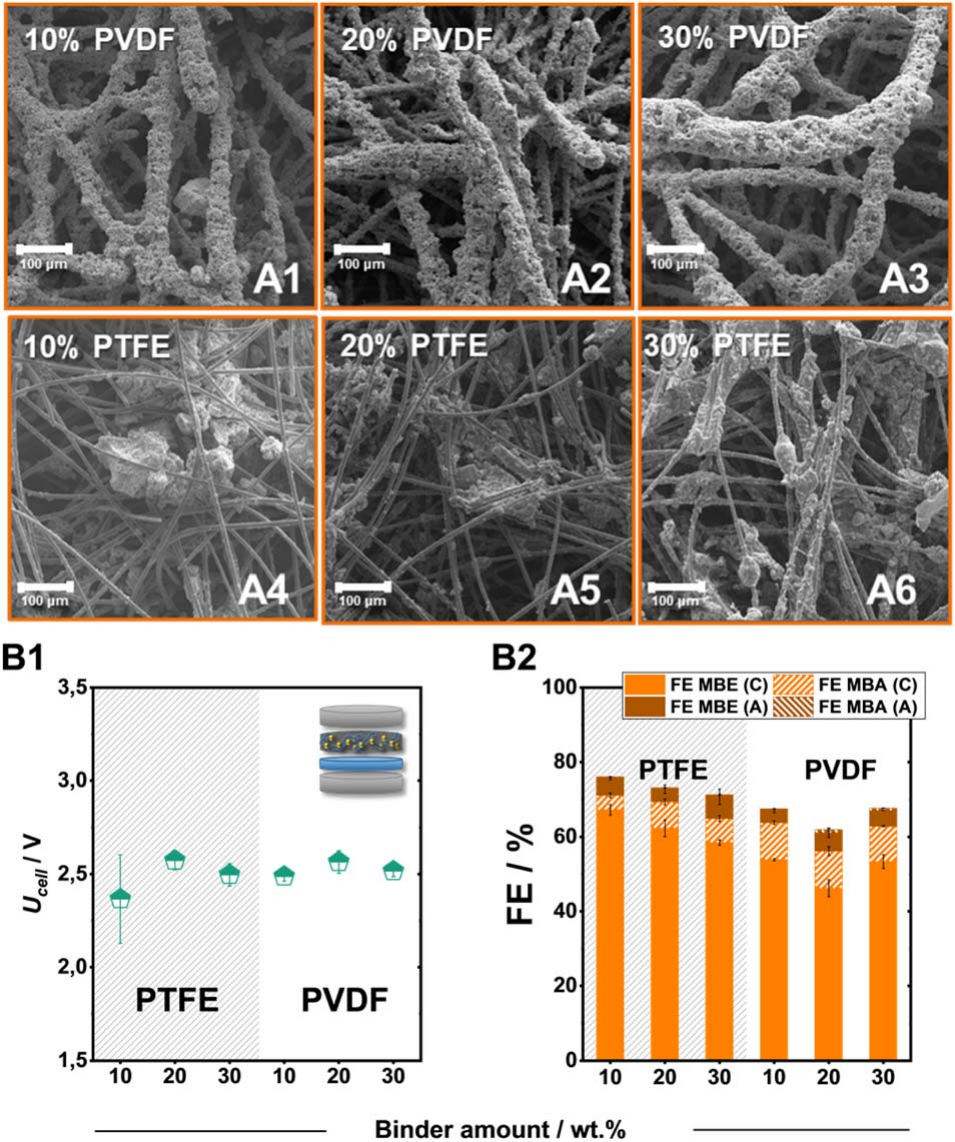
Scanning electron microscopy (SEM) investigation of PVDF (A1–A3) and PTFE bound electrodes (A4–A6). Effect of the binder nature and content on the ECH of MBY at 80 mA cm^−2^. The obtained cell voltage (B1) and faradaic efficiency (B2). Here, FE_MBX_ (C) and FE_MBX_ (A) (X: E, A) correspond to the FE values of ECH products detected in the catholyte and anolyte after electrolysis, respectively.

In addition to the carbon support, the employed binder also plays a key role in tailoring the local environment around the catalytic particles.^37^ Therefore, we altered the employed fluoropolymer binder from PTFE to PVDF as well as the added amount, to identify further tuning points. Overall, both PVDF and PTFE bound electrodes on SGL-GFD show FE values for the ECH of over 50%, with PTFE electrodes outcompeting their polymeric counterparts both in terms of activity for the ECH and specifically for the generation of MBE (Fig. 4B1 and B2). Despite the significantly different FE values, the generated electrodes demonstrate similar cell voltages of 2.5 V independently of the employed binder or added amount. In contrast to the employment of PTFE, which leads to the formation of catalytic cluster on the carbon felt fibres, the use of PVDF leads to a significant engulfment of the catalytic particles by the polymeric binder (Fig. 4A1–A6). The thickness of these PVDF-containing layers, as well as the size of the PTFE-formed clusters, appears to increase with the stepwise addition of the fluoropolymer binder. The observed particle agglomeration could also cause the lower FE values for the ECH at higher binder loadings, since a significant proportion of the catalytic material remains encased in these structures not contributing to the ongoing catalytic processes.

Further attempts to increase the energy efficiency of our process by employing elevated temperatures and thinner ion-exchange membranes did not severely alter the obtained values (Fig. S16†).^14,38^ At a cell temperature of 40 °C, the cell voltage decreases by approximately 0.5 V (2.0 V), resulting in similar FE values compared to ambient conditions. The stepwise increase of the operational temperature leads to a steady decrease of the overall ECH activity. This trend could be associated simultaneously with a limited adsorption of the organic substrate on the catalytic surface as well as improved kinetics for the HER under the elevated temperatures.^39^ Similarly, employing thinner anion-exchange membranes, of 50 μm and 30 μm thickness (as compared to 130 μm) did not yield any significant improvements in terms of cell voltage, while the total amount of ECH products crossing over to the anolyte increased from 4% (130 μm) to 13% (30 μm), attributed to electroosmosis and membrane swelling.^40^

### Pentlandite electrocatalysts *versus* the current state-of-the art

**Fig. 5:**
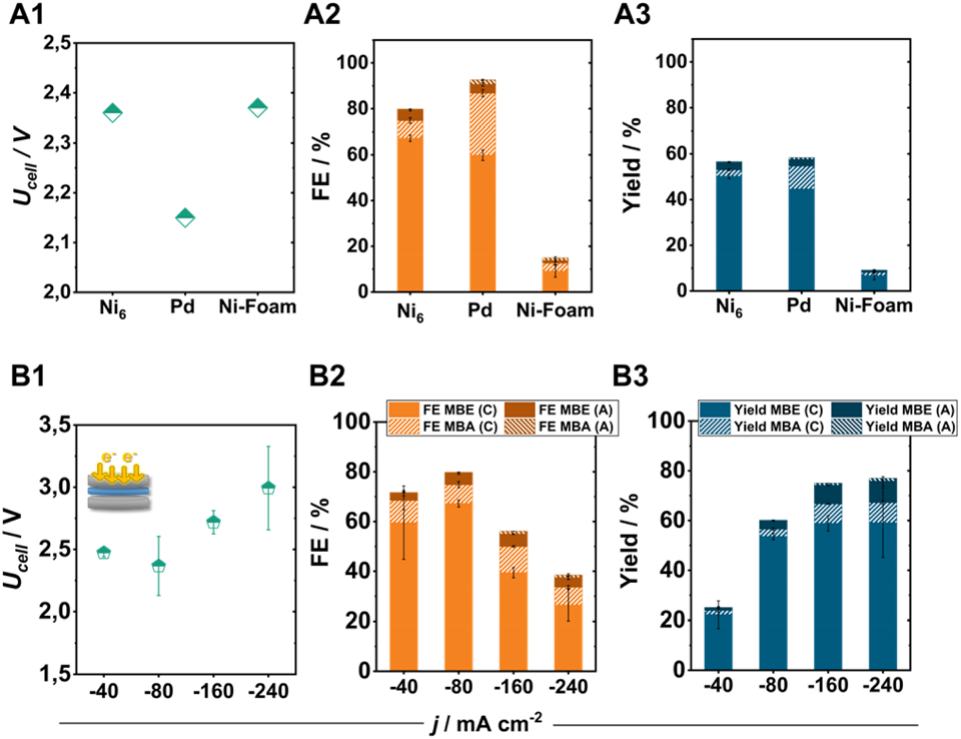
Comparison of Ni_6_ with the typical electrocatalysts for the ECH, Pd and Ni-foam. The obtained cell voltage (A1), faradaic efficiency (A2), and yield (A3) after 2 h of electrolysis at 80 mA cm^−2^. Effect of the applied current density on the ECH product distribution for Ni_6_-based electrodes in a zero-gap electrolyser. The obtained cell voltage (B1) faradaic efficiency (B2) and yield (B3) after 2 h of electrolysis at the applied current density. Here, FE_MBX_ (C) and FE_MBX_ (A) (X: E, A) correspond to the FE values of ECH products detected in the catholyte and anolyte after electrolysis, respectively.

Comparing the Ni_6_-coated electrodes with commonly used ECH catalysts such as Pd particles (0.35–0.8 μm) or Ni-foam under standard conditions further illustrates the high selectivity of pentlandite materials for the targeted semi-hydrogenation reaction (Fig. 5A1–A3). Specifically, identically prepared Pd-based electrodes show a higher tendency towards the hydrogenation of MBY to MBA (FE_MBA_: 30%) accompanied by a comparatively lower FE_MBE_ at similar MBY conversion. In contrast, the integration of Ni-foam as the cathode material demonstrates a decreased activity for the ECH with a total FE-ECH value of just 15%.^8^

By increasing the applied current density to achieve higher product yields, our optimized electrode was able to perform the ECH at elevated current densities of 240 mA cm^−2^, with a maintained FE_MBE_ of 30% and a FE_MBE_ : FE_MBA_ ratio of 7 : 1 (the remaining 70% FE belonging to the formation of H_2_). Even at these elevated current densities, the crossover of organic products to the anode compartment remains at 5%, allowing us to reach a total MBE yield of 70% in 2 h of electrolysis (Fig. 5B3) when combining the generated amounts of MBE in catholyte and anolyte. Regarding cell efficiency, the obtained cell voltages range from 2.4 to 3.0 V depending on the applied current density, demonstrating the capabilities of electrosynthetic anion-exchange-based MEAs to compete with the often PGM-containing cation-exchange variants.^41^ Subsequent long-term experiments for 100 h under elevated electrolytic loading, also demonstrate the high stability of pentlandite electrodes (Fig. S17†). Interestingly, although catalyst deactivation is a major issue in thermocatalytic processes, the herein demonstrated elevated stability indicates that ECH reactors could possibly possess a longer lifetime than their counterparts by minimization of by-product formation.

Overall, the observed activity constitutes currently the only example of performing hydrogenation of MBY in aqueous electrolytes without the involvement of any PGM-metals in either cell compartment (Table S3†).

Despite the highly competitive performance of our combined catalyst and electrode optimization in the field of ECH, there is yet still significant room for optimization in direct comparison to thermocatalytic processes. In view of the present results, however, we are confident that the community will be able to establish a green and economically competitive alternative to thermocatalytic processes soon.

## Conclusions

In conclusion, we demonstrate the electrochemical reduction of the alkynol 2-methyl-3-butyne-ol (MBY) to its commercially important semi-hydrogenation product 2-methyl-3-butene-ol (MBE) under industrial relevant conditions using a non-noble metal catalyst, without the supply of H_2_ gas, in aqueous electrolytes. This important milestone was achieved using cheap and robust transition metal chalcogenides based on the mineral pentlandite (M_9_S_8_), further demonstrating the multi-functionality of this material class in a range of reactions such as the ECH, HER, CO_2_RR and OER. Moreover, we herein provide a rare example of the utilization of a PGM-free zero-gap electrolyser in an aqueous-based organic reaction. *Via* optimization of the carbon support and binder for the ECH, we were able to reach ECH yields of 70% for the conversion of MBY to MBE after 2 h of electrolysis at 240 mA cm^−2^ at cell voltages as low as 3.0 V. We believe that our catalytic and cell parameter screening provide important stepping-stones towards the development of more financially favourable, active and sustainable electrocatalysts for the ECH of substrates beyond alkynols as well as towards the acceleration of this technology in the industrial scale.

## Author contributions

Conceptualization: K. P., J. T. K., K. J. P., D. S. and U.-P. A.; methodology: K. P., L. W., J. T. K., K. J. P., D. S. and U.-P. A.; data curation: K. P., L. W., J. W., A. L. and D. T.; formal analysis: K. P., L. W., J. W., A. L. and D. T.; funding acquisition: U.-P. A. and D. S.; supervision: U.-P. A., K. J. P. and D. S.; writing-original draft preparation: K. P., L. W., J. T. K., R. C., J. M., R. G., U.-P. A. and D. S. All authors have read and agreed to the published version of the manuscript.

## Data availability

Experimental data regarding electrochemical experiments and product analysis are available free of charge in the ESI.†

## Conflicts of interest

These are not conflicts of interest to declare.

## Supplementary Material

SC-013-D2SC04647D-s001
