# Supplementary material for: Opening the pathway towards a scalable electrochemical semi-hydrogenation of alkynols via earth-abundant metal chalcogenides
Source: Chem Sci. 2022 Oct 11;13(42):12461–8. doi: 10.1039/d2sc04647d (PMC9629083; doi:10.1039/d2sc04647d)
Supplement: SC-013-D2SC04647D-s001 [file SC-013-D2SC04647D-s001.pdf]

Electronic Supplementary Material (ESI) for Chemical Science.
This journal is © The Royal Society of Chemistry 2022

Supporting Information

Opening the Pathway Towards a Scalable Electrochemical Semi-Hydrogenation of Alkynols via Earth-Abundant Metal Chalcogenides

Kevinjeorjios Pellumbi[a,b]†, Leon Wickert[a,b]†, Julian T. Kleinhaus,[b] Jonas Wolf[a,b], Allison Leonard[b], David Tetzlaff[a,b], Roman Goy,[c] Jonathan A. Medlock,[c] Kai junge Puring[a], Rui Cao[d], Daniel Siegmund[a,b]* and Ulf Peter Apfel[a,b]*

a. Fraunhofer Institute for Environmental, Safety and Energy Technology UMSICHT, Osterfelder Straße 3, DE-46047 Oberhausen, Germany.

b. , Inorganic Chemistry I, Ruhr University Bochum Universitätsstraße 150, 44780 Bochum, Germany.

c. DSM Nutritional Products AG, Wurmisweg 576, CH-4303 Kaiseraugst, Switzerland.

d. Key Laboratory of Applied Surface and Colloid Chemistry, Ministry of Education, School of Chemistry and Chemical Engineering, Shaanxi Normal University, Xi'an 710119, China.

**Correspondence: ulf.apfel@umsicht.fraunhofer.de, ulf.apfel@rub.de and daniel.siegmund@umsicht.fraunhofer.de*

† These authors contributed equally

Table of contents

Materials 3

Mechanochemical Synthesis of the $Fe_{9-x}Ni_xS_8$ & MCo_8S_8 pentlandite catalysts 3

Physical Characterization 3

PXRD Analysis 4

Electrochemical investigation of pellet electrodes in a PEEK H-type cell 5

Product quantification for the ECH with pellet electrodes 5

$Fe_3Ni_6S_8$ *vs.* MCo_8S_8 pentlandites for the hydrogenation of MBY 6

$Fe_3Ni_6S_8$ *vs.* mono-metallic sulphides for the hydrogenation of MBY 6

Comparison of the different pentlandite catalyst towards selectivity for MBE *vs.* MBA 7

Electrochemical investigations in a zero-gap electrolyzer 8

Electrode Fabrication 9

NMR Analysis 10

Quantification of generated hydrogen via GC-MS 12

H_2 Quantification during electrolysis in the zero-gap electrolyzer 12

Product quantification for the zero-gap experiments 13

Properties & Vendors of the different employed GDLs 14

SEM Analysis of the tested electrodes in the zero-gap electrolyzer 15

EDX Analysis of the tested electrodes 16

Effect of temperature and membrane thickness on the ECH activity 19

Hydrogenation of MBY: Electrocatalysis vs. Thermocatalysis 20

Hydrogenation of MBY: Conversion factors & Reaction rates 21

Long-term ECH experiments with pentlandite-based electrodes 23

References 24

Materials

Unless otherwise stated, all chemicals were purchased from commercial vendors and used without further purification. The elemental iron, nickel, cobalt and sulphur used for the synthesis of the different pentlandites were purchased from Alfa Aesar. NiS and FeS were kindly provided by Tribotecc GmbH. 2-methyl-3-butyn-2-ol (98 %), 2-methyl-3-buten-2-ol (98 %) and 2-methyl-3-butan-2-ol (98 %) were purchased from Sigma Aldrich.

Mechanochemical Synthesis of the $Fe_{9-x}Ni_xS_8$ & MCo_8S_8 pentlandite catalysts

The synthesis of the catalysts was conducted through mechanochemical means.[1] The Pn-materials were synthesized from the high-purity elemental powders. Under argon atmosphere, 5 g of a stoichiometric mixture of the elemental powder and 24 g of 2 mm zirconium oxide milling balls were mixed in a 20 mL zirconium oxide grinding vessel. In a Fritsch Planetary Micro Mill Pulverisette 7 premium line, the mixture was milled 120 min at 1100 rpm for two cycles with a 60 min break in between to obtain the pentlandite catalyst.

Physical Characterization

SEM imaging was either performed using a TESCAN Vega 3 microscope or a ZEISS Gemini2 Merlin HR-FESEM equipped with an OXFORD AZtecEnergy X-ray microanalysis system for energy dispersive X-ray spectroscopy (EDX).

PXRD Analysis

Powder X-ray diffraction (PXRD) measurements were performed on a Bruker D2 Phaser diffractometer equipped with a LynxEye detector operating at 30 kV acceleration voltage and 10 mA emission current using Cu K-α radiation (λ = 1.54184 Å). The data was recorded in a range from 10–70° 2θ.

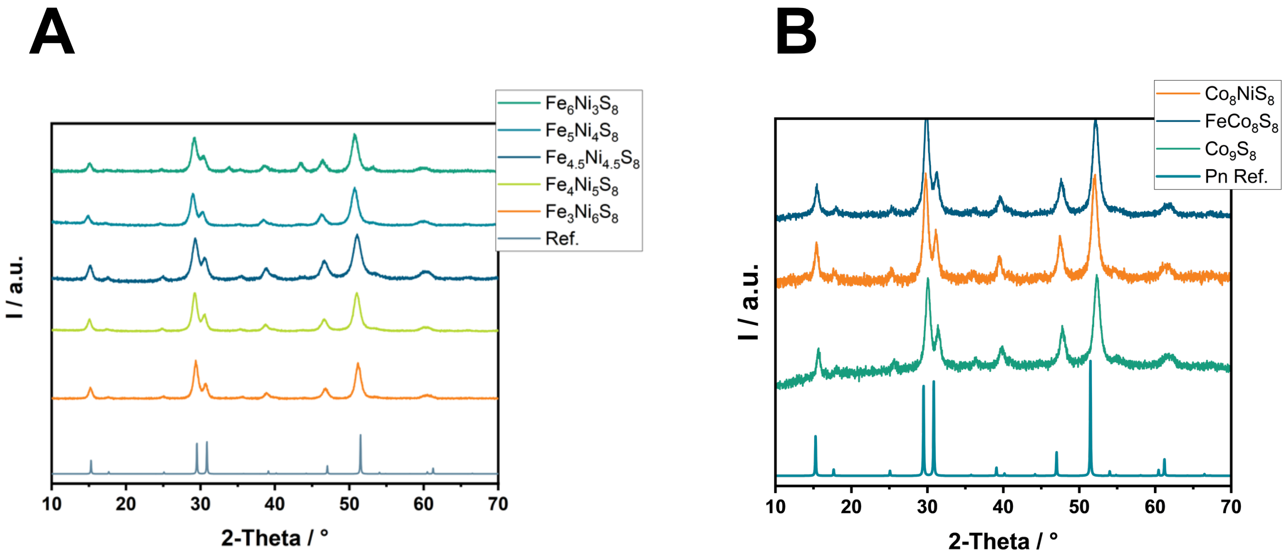

Figure S1. Powder X-ray diffractograms of the obtained Ni/Fe ratios (A) and MCo_8S_8 (B) pentlandite materials through the mechanochemical synthesis.

Electrochemical investigation of pellet electrodes in a PEEK H-type cell

Fabrication of the employed electrode was performed similarly to previous investigations.[2] In short, approximately 50 mg of the obtained powder materials was pressed with a minimum pressure of 800 kg cm^{-2} into a 3 mm pellet with a geometric area of 0.071 cm^2. The obtained pellets were integrated into electrodes consisting of a brass rod with screw threads and a PTFE housing. As a binder material carbon glue (3M) was employed. The prepared electrodes were placed in an oven overnight at 60°C to allow the glue to harden. Afterwards, the use of 12 µm, 3 µm and 0.3 µm sanding papers to polish the pellet ensured an even, smooth surface.

To remove any oxide species from the surface of the electrode, conditioning of the electrode was performed prior to electrolysis via linear sweep voltammetry. Specifically, a potential range from 0 to -0.5 V vs. RHE at a scan rate of 100 mV s^{-1} was applied, till a stable electrochemical response in the voltammogram was obtained. Electrolysis was performed under galvanostatic conditions at 100 mA cm^{-2} for 2 h under constant stirring of the electrolyte. A Ni-mesh served as the counter electrode, while a RHE (MiniRHE from Gaskatel) was used as the reference electrode. The anode compartment was filled with 12 mL of 1 M KOH, while the cathode compartment contained 12 mL of 1 M MBY in 0.3 M KOH/H_2O. The two half-cells were separated with the help of a Nafion117 cation exchange membrane. Each measurement was repeated at least two times.

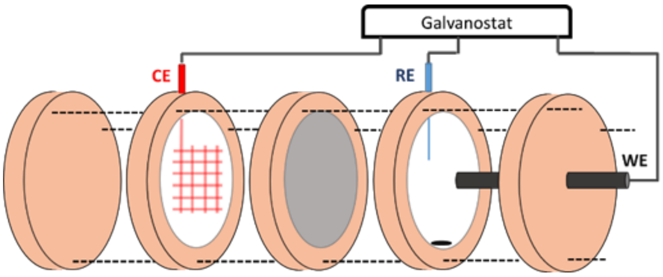

Figure S2. Schematic representation of the herein employed PEEK H-type cell for the screening of pellet electrodes.

Product quantification for the ECH with pellet electrodes

Analysis of the electrolysis products in the PEEK H-type cell was performed via headspace GC-MS analysis. The quantification took place by directly analyzing 1 mL of the obtained catholyte utilizing a Shimadzu GC-MS QP2020 equipped with a HS20 headspace sampler. Calibration was performed using external standard solutions consisting of MBE & MBA in 0.3 M KOH in H_2O, as well as blank MBY solutions.

$Fe_3Ni_6S_8$ *vs.* MCo_8S_8 pentlandites for the hydrogenation of MBY

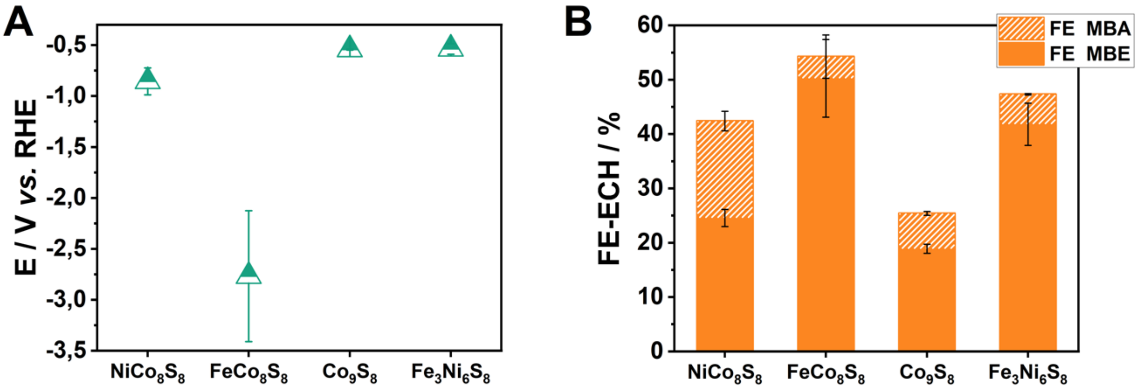

Figure S3. Obtained half-cell potentials after 2 h of electrolysis at -100 mA cm^{-2} of the different pentlandites employing pellet electrodes in a H-type cell (**A**). Respective faradaic efficiency for the ECH quantified via GC-MS (**B**).

$Fe_3Ni_6S_8$ *vs.* mono-metallic sulphides for the hydrogenation of MBY

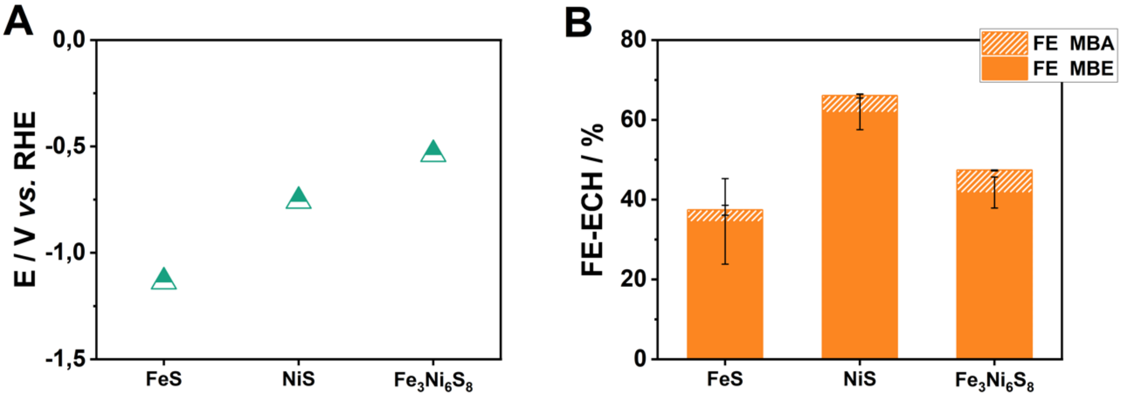

Figure S4. Obtained half-cell potentials after 2 h of electrolysis at -100 mA cm^{-2} of different transition metal sulphides employing pellet electrodes in a H-type cell (**A**). Respective faradaic efficiency for the ECH quantified via GC-MS (**B**).

Comparison of the different pentlandite catalyst towards selectivity for MBE *vs.* MBA

Table S1. Comparison of the different pentlandite catalysts regarding the selectivity towards the generation of MBE vs. MBA from a pellet electrode screening.

Catalyst	FE_{MBE} / %	FE_{MBA} / %	FE_{MBE} / FE_{MBA}	E / V vs. RHE
$Fe_3Ni_6S_8$	42	5.5	7.6	-0.54
$Fe_4Ni_5S_8$	40	9.4	4.2	-0.66
$Fe_{4.5}Ni_{4.5}S_8$	28	4.8	5.8	-0.70
$Fe_5Ni_4S_8$	34	2.5	13.5	-0.50
$Fe_6Ni_3S_8$	28	3.8	7.3	0.52
Co_9S_8	18	7	2.9	-0.54
$FeCo_8S_8$	50	4	12.5	-2.7
$NiCo_8S_8$	25	17	1.4	-0.85

Electrochemical investigations in a zero-gap electrolyzer

Electrochemical investigations in membrane electrode assemblies were performed in an in-house built single cell electrolyzer with an electrode area of 12.57 cm^2 (40 mm diameter). A compressed Ni-foam served as the anode, a porous carbon electrode coated with 5 mg cm^{-2} of the respective catalysts served as the cathode, employing PTFE gaskets and a torque value of 5 N m over eight screws to ensure a leak-less operation. The used Fumasep membranes were conditioned overnight in 1 M KOH before use. During electrolysis, the anolyte and catholyte solutions were circulated through the half cells at flow rate of 12 ml min^{-1}, while a homogeneous liquid distribution was guaranteed through the use of Ti-based serpentine flow fields. A copper plate in direct contact with the flow field served as the current collector plate. Electrolysis was performed for 2 h, with samples being taken at the end of electrolysis from both the anolyte and catholyte. The respective chronopotentiometric curves up to a current density of 80 mA cm^{-2} (1 A) were recorded on a Gamry Interface 1010B potentiostate/galvanostate, while for current density values above 80 mA cm^{-2}, a Gamry Reference 3000 potentiostate/galvanostate equipped with a 30K booster module was employed. For each experiment, a new MEA was constructed/investigated, with each experiment being investigated at least two times.

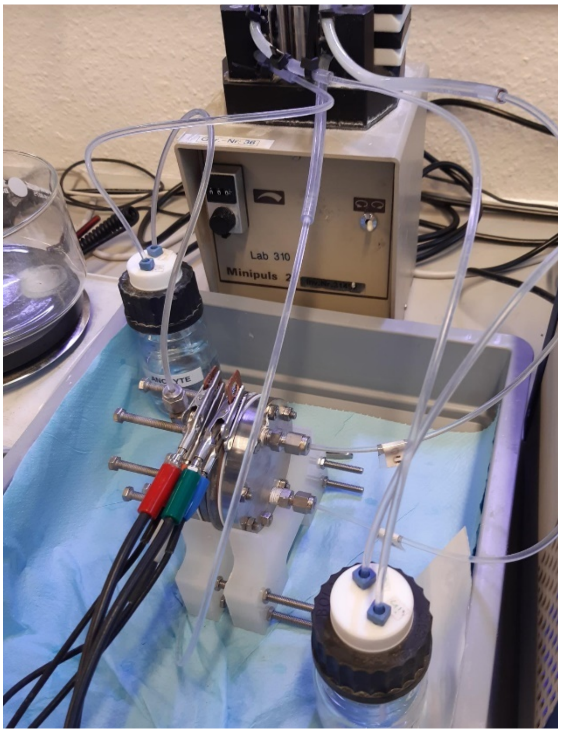

Figure S5 Exemplary picture of the employed zero-gap electrolyzer set-up used in this work.

Electrode Fabrication

To prepare the catalyst PTFE-containing catalyst inks, 0.5 g of the mechanochemically synthesized $Fe_3Ni_6S_8$ catalyst was mixed with 15 g 2-propanol, 4 mL H_2O and 0.2 g Triton X 100. The mixture was placed into an ultrasonic bath for 5 min. Afterwards, the ink was dispersed at 13600 rpm using a T 25 digital Ultra-Turrax for 1 min. Afterwards, the respective amount of a 60 wt% PTFE dispersion was added while stirring. The suspension was then homogenously spray-coated with an Iwata SBS airbrush on a 8.5x8.5 cm carbon cloth or carbon felt that was heated on a hot plate to 100 °C. Afterwards, the PTFE-containing electrodes were heat-treated at 240 °C for 20 min to remove the surfactant in the ink.

For the PVDF containing ink, a 5 wt% PVDF solution in methanol/acetone was added in a solution consisting of 0.5 g $Fe_3Ni_6S_8$ in 4 mL H_2O and 15 g of methanol, to reach the desired amount of binder. The ink was homogenously spray-coated with an Iwata SBS airbrush on a 8.5x8.5 cm carbon cloth or carbon felt that was heated on a hot plate to 100 °C. No further treatment steps were performed in the case of PVDF-containing inks.

The resulting catalyst coated sheets were cut into circular electrodes of a diameter of 40 mm with the help of an iron punch. The catalytic loading was determined through the weight diffrence between the non-coated substrates and dried coated electrodes.

NMR Analysis

The catholyte and anolyte solution samples were additionally analyzed via ^{1}H-NMR spectroscopy with potassium hydrogen phthalate as internal standard. NMR samples consisted of 50 µl sample solution, 50 µl of a solution containing 1.25 µmol potassium hydrogen phthalate standard in D_2O and 400 µl D_2O as solvent. ^{1}H-NMR analysis was conducted using an Avance-III 300 MHz spectrometer at 22 °C. Under the applied conditions and concentrations, equal integrals of the standard and the product protons correspond to equal concentrations as confirmed by control experiments with known amounts of MBY, MBE and MBA.

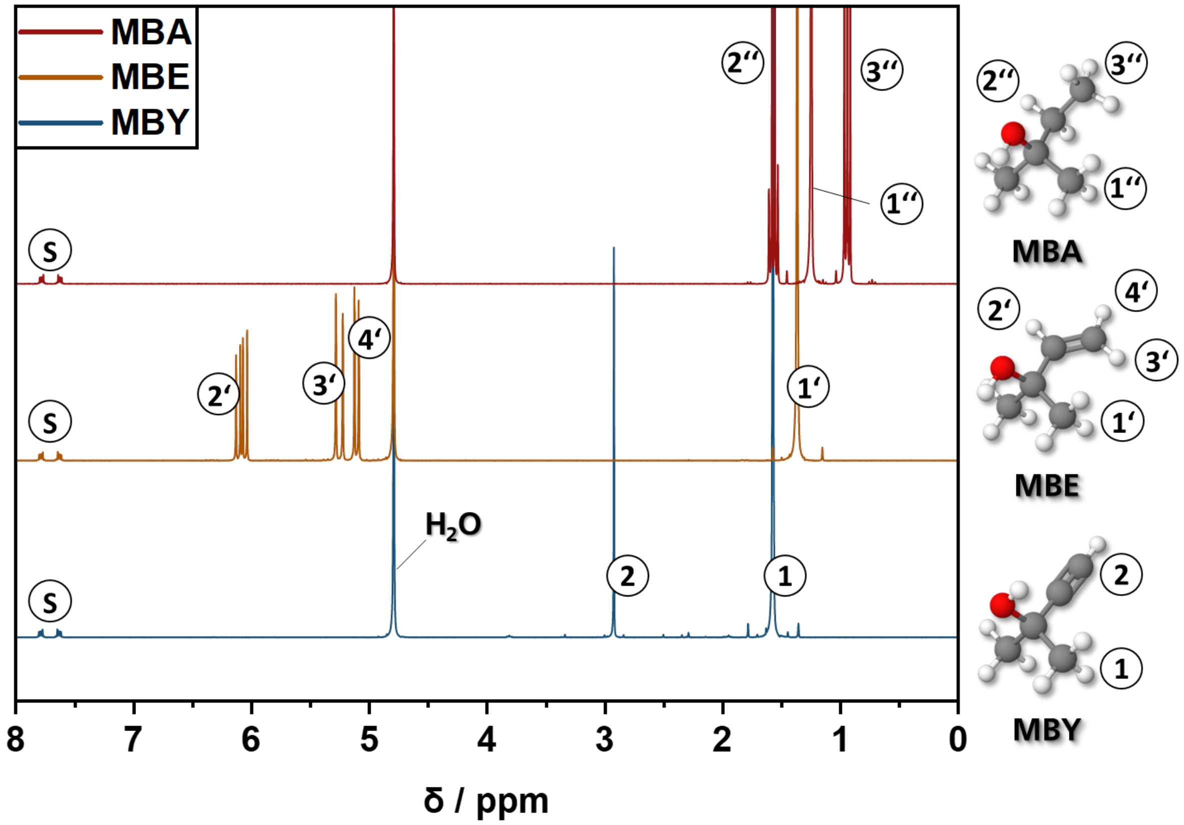

Figure S6. ^{1}H-NMR spectra of 1 M MBY, MBE and MBA, respectively with addition of the employed NMR standard (S).

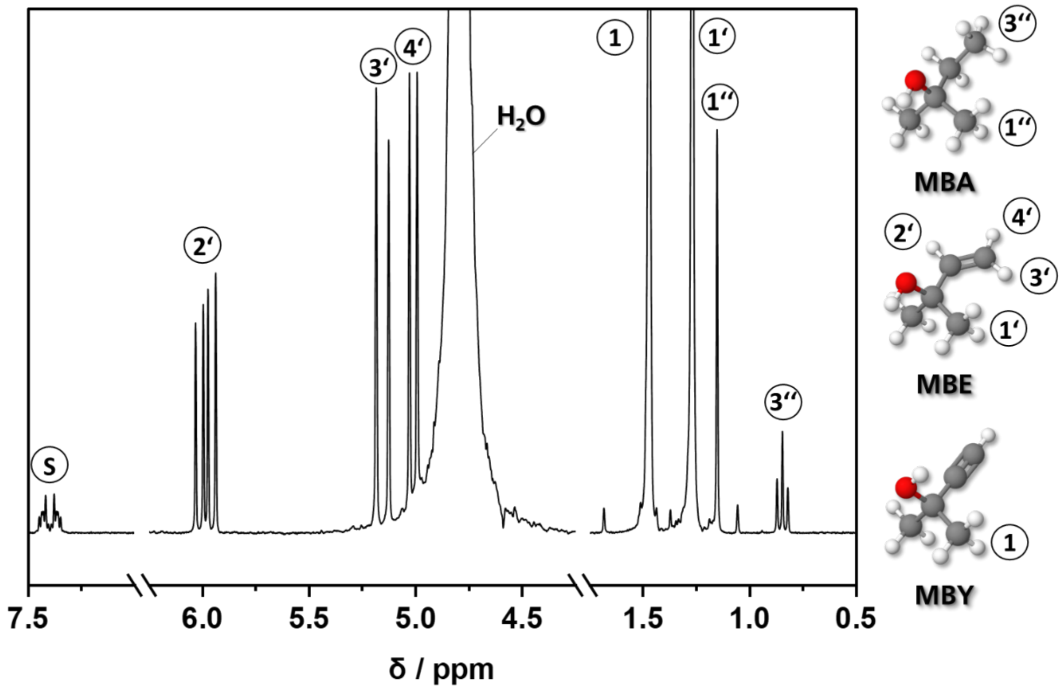

Figure S7. Representative ¹H-NMR spectrum of the NMR sample of the catholyte solution after the ECH with 5 mg cm^{-2} $Fe_3Ni_6S_8$ on graphite felt at -80 mA cm^{-2} with an AEM and the addition of a quantification standard (S).

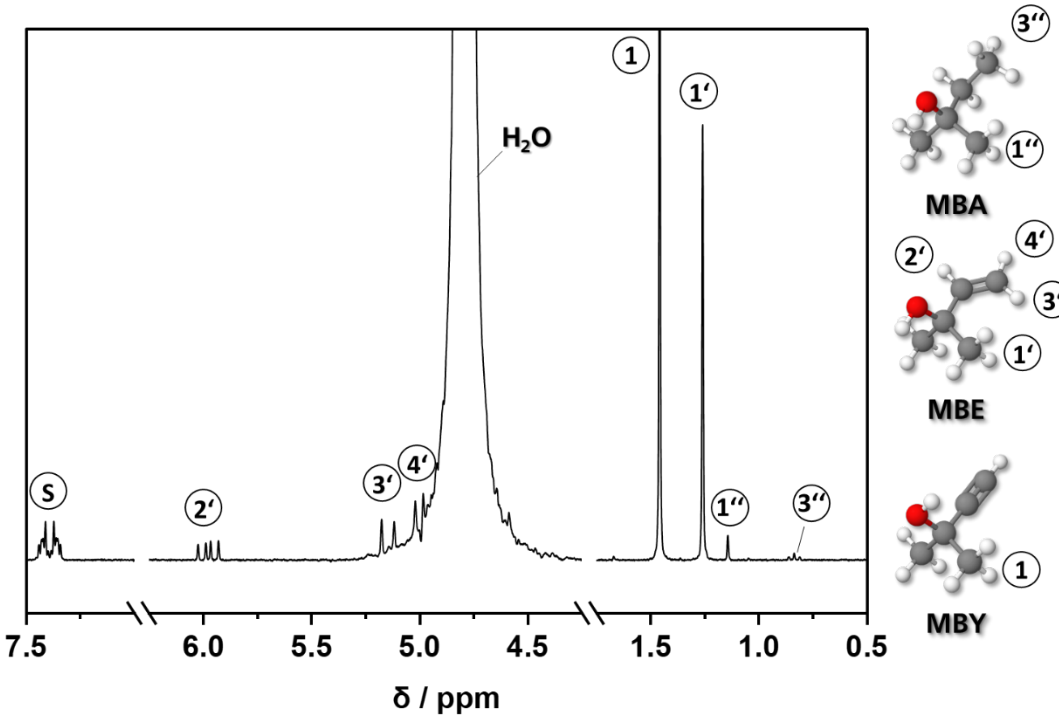

Figure S8. Representative ¹H-NMR spectrum of the NMR sample of the anolyte solution after the ECH with 5 mg cm^{-2} $Fe_3Ni_6S_8$ on graphite felt at -80 mA cm^{-2} with an AEM and the addition of a quantification standard (S).

Quantification of generated hydrogen via GC-MS

Quantification of the generated hydrogen during ECH in zero-gap electrolyzers was performed with the help of an online Shimdazu QP2020 GC-MS equipped with a Supelco Carboxen 1010 Plot column. The catholyte reservoir was completely sealed with Ar flowing through at a flow rate of 10 ml min^{-1}. Gas-samples were taken every 20 min for a total of 2 h.

H_2 Quantification during electrolysis in the zero-gap electrolyzer

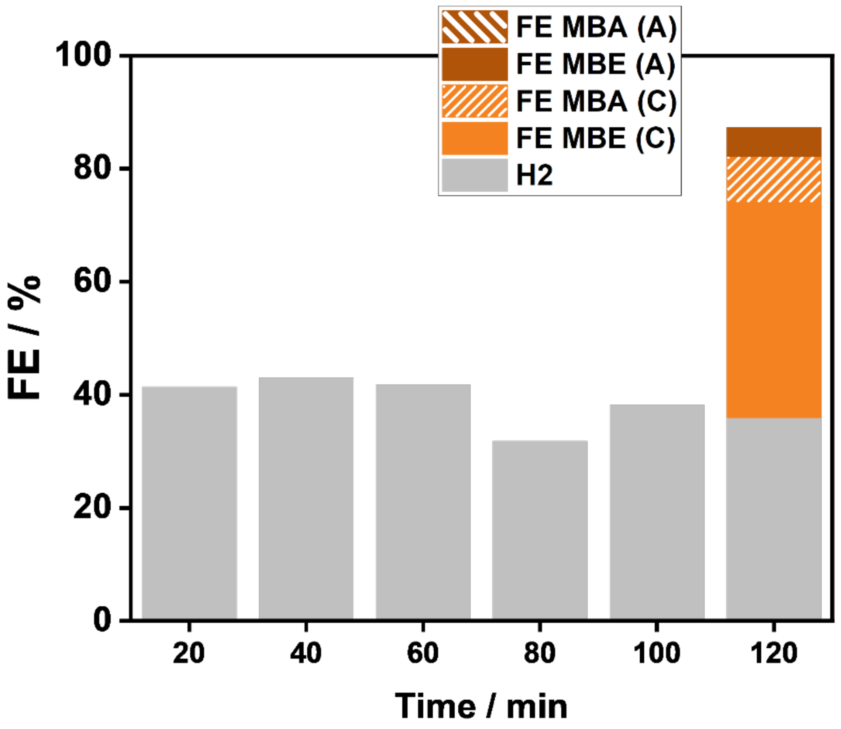

Figure S9. Total faradaic efficiency during electrolysis at 80 mA cm^{-2} for 2 h. Here FE_{MBX}(C) and FE_{MBX}(A) (X: E, A) correspond to the FE values of ECH products detected in the catholyte and anolyte after electrolysis, respectively.

Compared to our experiments under ambient conditions, during the H_2 quantification the total FE for the ECH decreases from ca 80 % to 55 %. This decrease could be attributed to the operation of the cell under the slight differential pressure of 40 mbar, which could have affected the fluid dynamics of the flow during electrolysis.

Moreover, from our control experiment we were able to obtain a total faradaic efficiency of 90 % including the generated H_2, and ECH products in the anolyte and catholyte. Since our NMR analysis show no signs of decomposition or side-products, we are confident that the remaining percentages could be attributed to small leaks within the electrolyzer or gas-sampling loop, since our ECH electrolyzer has not been yet optimized towards a completely leakfree operation for gaseous products during electrolysis.

Product quantification for the zero-gap experiments

The faradaic efficiency of MBE and MBA was calculated with equation 1 (with n_p as amount of product p in mol, z as number of transferred electrons ($z = 2$ for MBE, $z = 4$ for MBA), F as Faraday constant (96485 A s mol^{-1}), i as applied absolute current in A and t as reaction time (7200 s):

$$F.E._p = n_p \cdot \frac{zF}{i \cdot t} \cdot 100\ \% \tag{1}$$

The amount of product n_p was calculated with equation 2 (with I as the integral of the product peak (6H) in the ^{1}H-NMR spectrum normalized to the peak integral of the internal standard (4H), and n_{St} as the amount of internal standard in the NMR sample (1.25 µmol)):

$$n_p = I \cdot {}^2/_3 \cdot n_{St} \cdot 1000 \tag{2}$$

The yield was determined with equation 3:

$$yield = \frac{n_p}{n_{MBY}} \cdot 100\ \% \tag{3}$$

FE and yield error bars were calculated from the results of duplicates.

The potential values have been calculated by determining the arithmetic mean of the respective potential data for all data points of the last 10 min of the experiment for both of the two measurements. The arithmetic mean and the standard deviation of the resulting values was determined as the final data point of the respective experiment.

Properties & Vendors of the different employed GDLs

Table S2. Listing of the different properties of the herein employed PTLs for the ECH of MBY in zero-gap electrolyzers.

PTL	Thickness / mm	Area weight / g m^{-2}	Properties	Vendor
H23	0.210	95	Carbon Paper<br>No additional treatment	Freudenberg
H23I2	0.222	114	Carbon Paper<br>Hydrophobic treatment	Freudenberg
W1S1010	0.365	175	MPL<br>Hydrophobic treatment	CeTech
SGL-GFD	2.5	250	Graphite Felt<br>No additional treatment	SGL<br>Carbon

SEM Analysis of the tested electrodes in the zero-gap electrolyzer

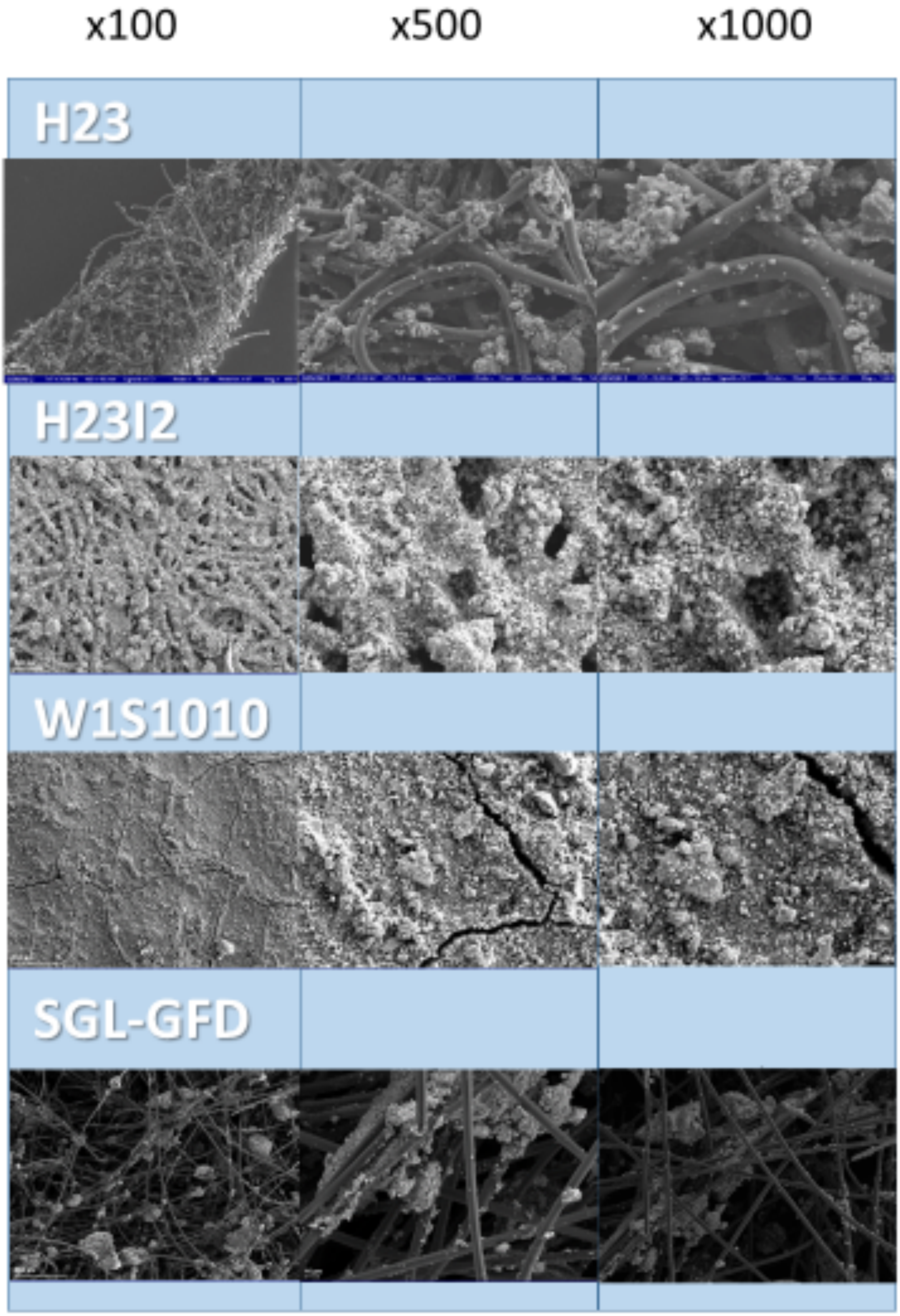

Figure S10. SEM Analysis of the catalyst coated PTLs at magnifications of x100, x500, x1000.

EDX Analysis of the tested electrodes

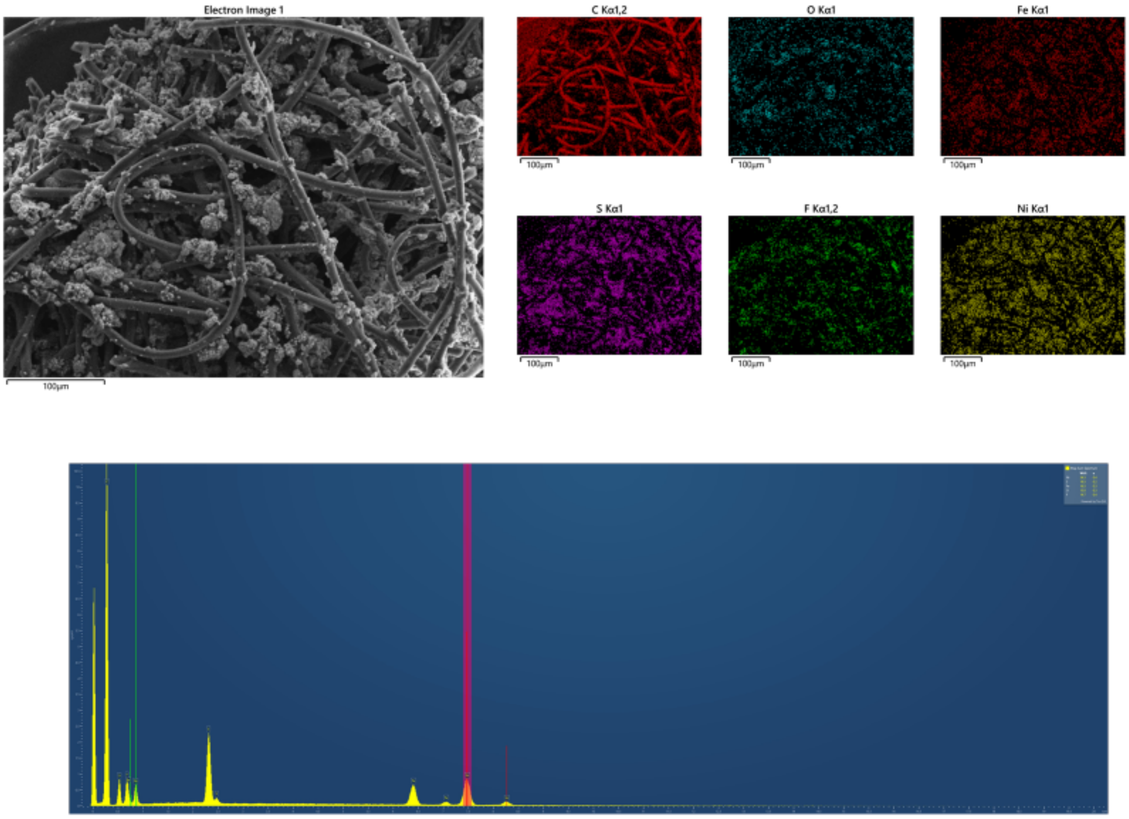

Figure S11. EDX analysis of a H23 coated PTL with 5 mg cm^{-2} of catalytic loading.

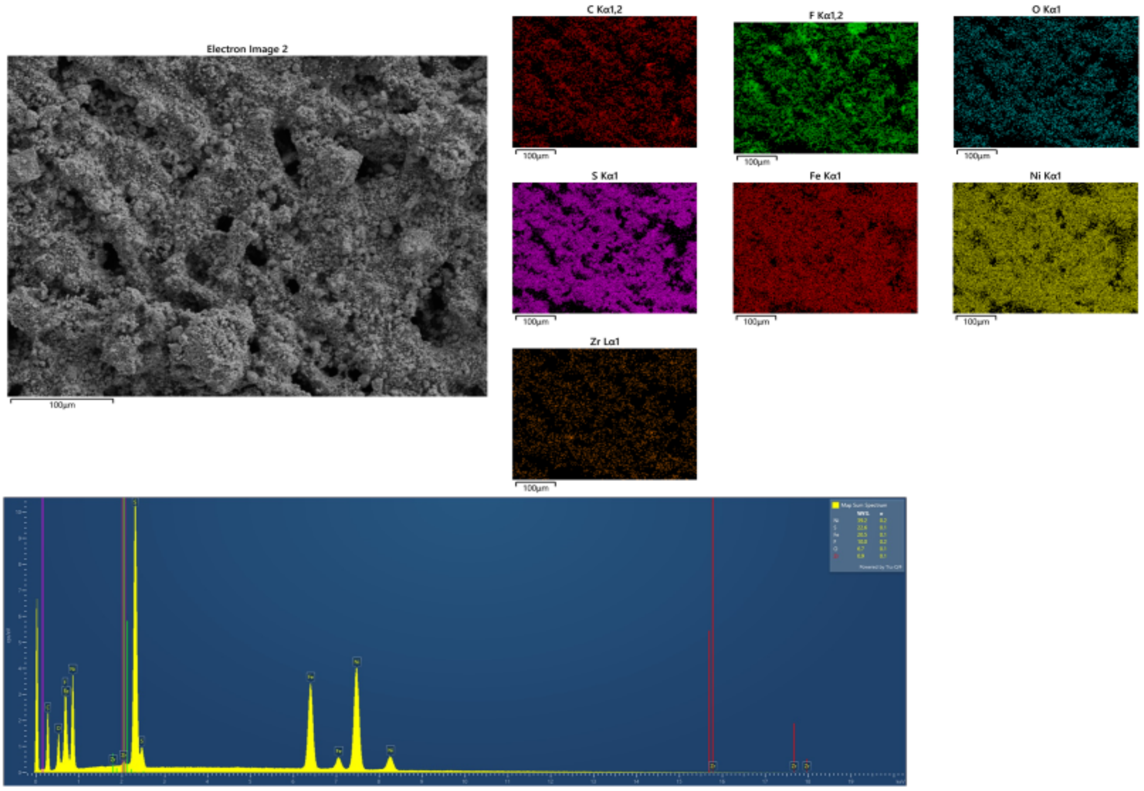

Figure S12. EDX analysis of the H23I2-PTL coated with 5 mg cm^{-2} of catalytic loading.

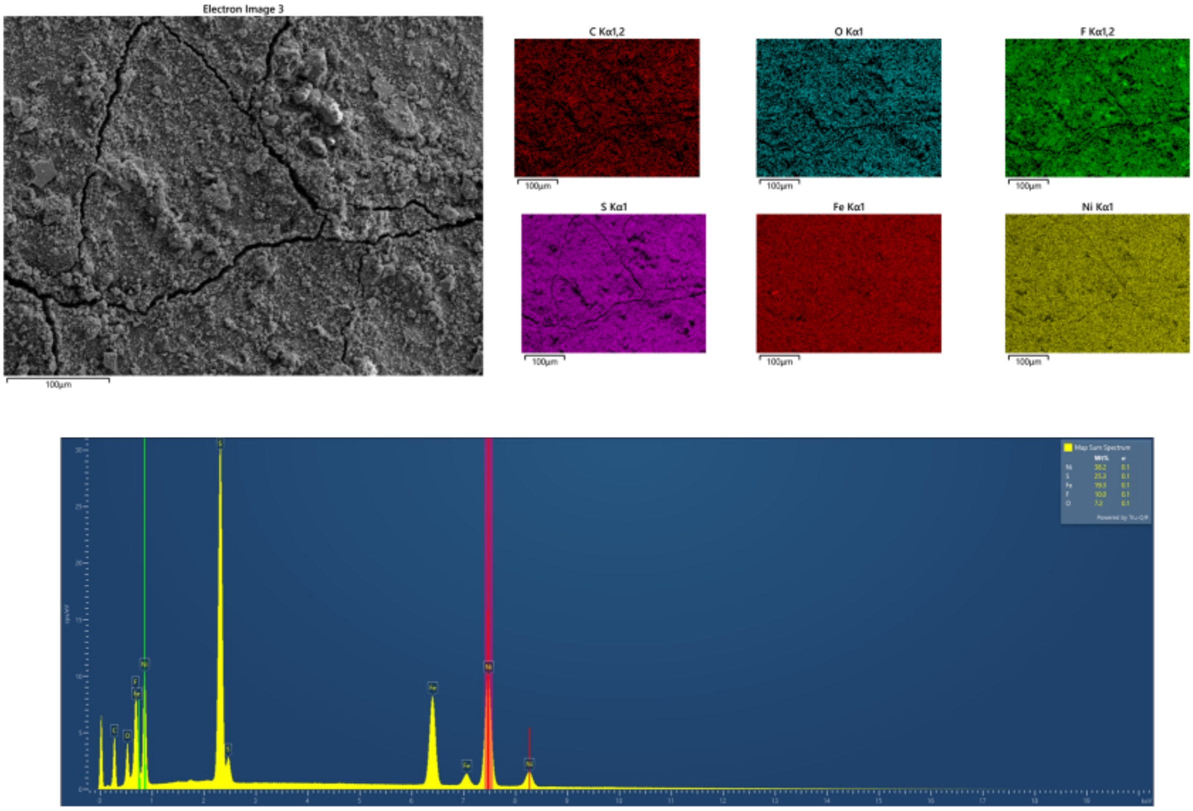

Figure S13. EDX analysis of the W1S1010- PTL coated with 5 mg cm^{-2} of catalytic loading.

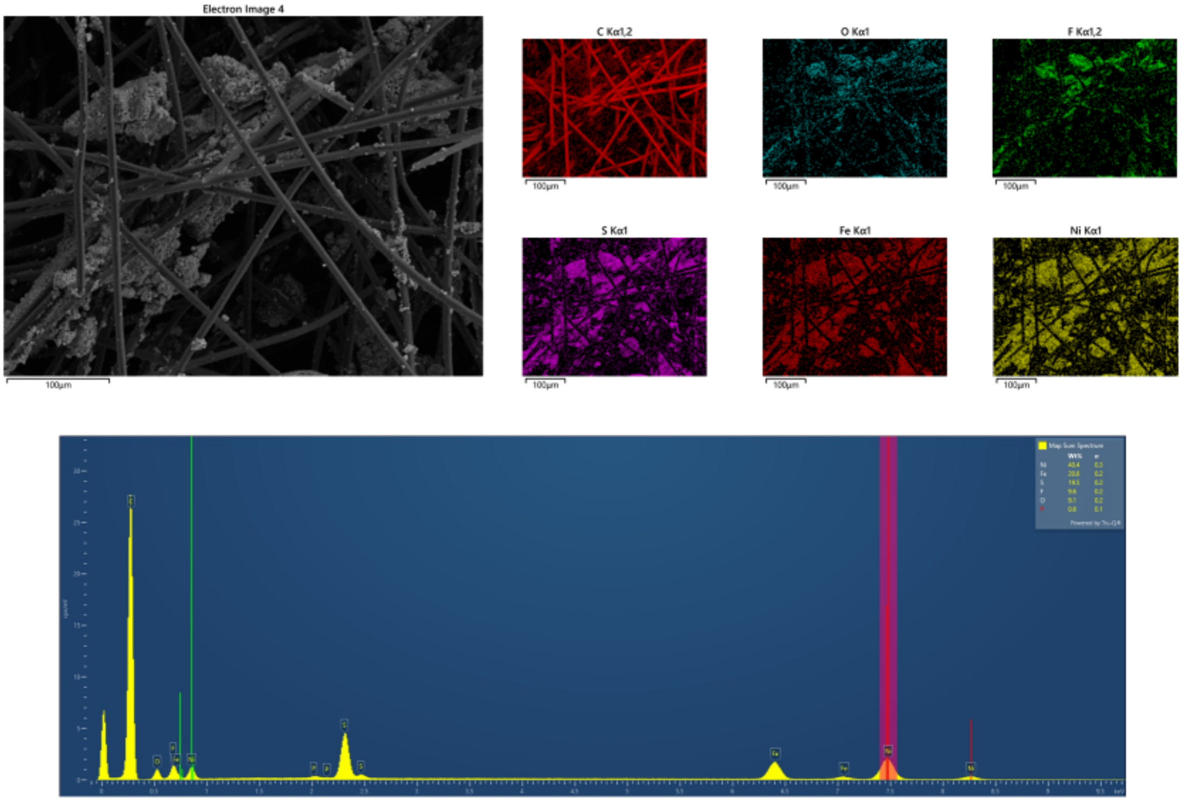

Figure S14. EDX analysis of the SGL-GFD coated PTL with 5 mg cm^{-2} of catalytic loading.

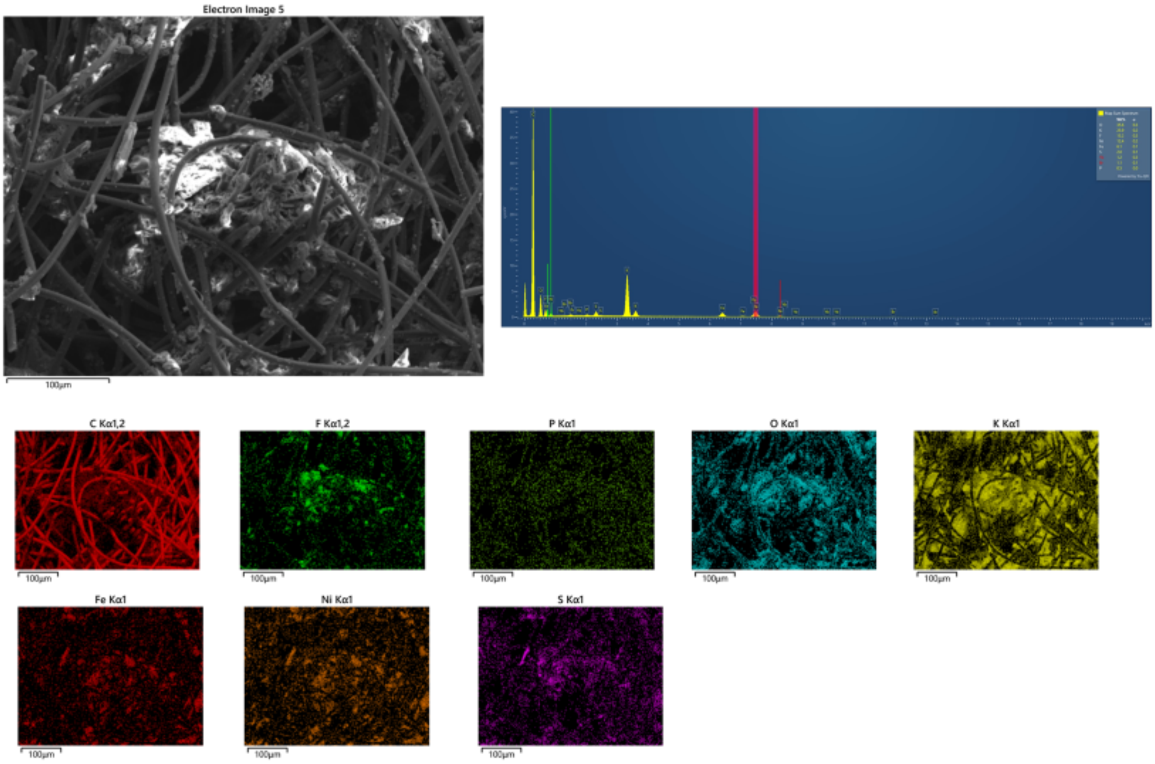

Figure S65. EDX analysis of a SGL-GFD coated PTL with 5 mg cm^{-2} of catalytic loading after electrolysis.

Effect of temperature and membrane thickness on the ECH activity

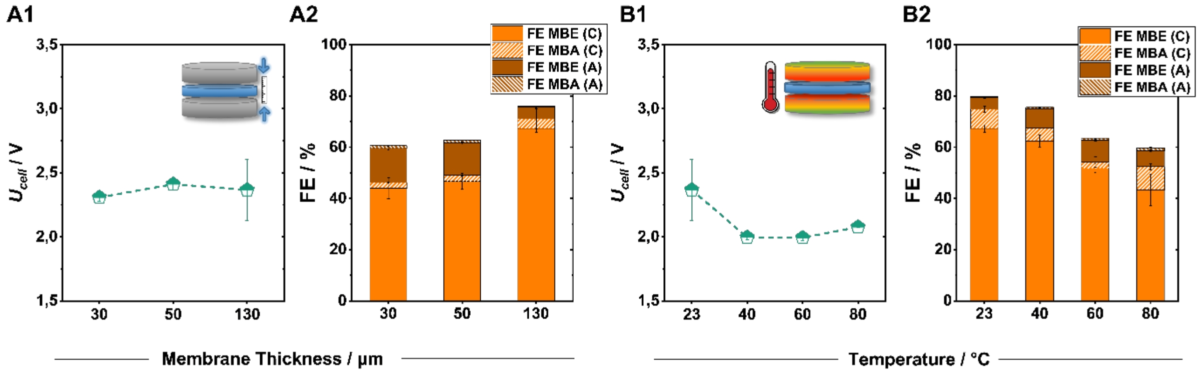

Figure S16. A) Investigation of the role of the employed AEM thickness for the hydrogenation of MBY at 80 mA cm^{-2} in 0.3 M KOH for 2 h of electrolysis, at a flow rate of 12 ml ml^{-1} in batch-mode. The obtained cell voltage (A1) and faradaic efficiency (A2) obtained through NMR quantification. B) Investigation of the role of the electrolyzer temperature for the hydrogenation of MBY at 80 mA cm^{-2} in 0.3 M KOH for 2 h of electrolysis, at a flow rate of 12 ml ml^{-1} in batch-mode. The obtained cell voltage (B1), faradaic efficiency (B2) through NMR quantification. Here FE_{MBX}(C) and FE_{MBX}(A) (X: E, A) correspond to the FE values of ECH products detected in the catholyte and anolyte after electrolysis, respectively.

Hydrogenation of MBY: Electrocatalysis vs. Thermocatalysis

Regarding the comparison with the current state-of-the-art systems, it is important to notice that most thermocatalytic reactors show a sharp increase in MBA generation after reaching a peak for the MBE conversion. Despite this finding, we specifically selected these peak values for the comparison with our electrochemical route.

Table S3. Comparison of the current state-of-the-art systems found in literature for the hydrogenation of MBY via thermocatalytic means against the herein present electrocatalytic route.

Catalysis	c(MBY)	Conversion MBY	Selectivity MBE	Catalyst	Conditions	Operation mode	Ref.
Thermal	solvent-free	99	91	Lindlar	333 K, 7 bar H_2	Batch	3
Thermal	solvent-free	97	99	Pd/ZnO	333 K, 7 bar H_2	Batch mode	3
Thermal	solvent free	95 after 100 min	95	Pd/ZnO/SMF	348 K, 8 bar H_2	Semi-batch mode	4
Thermal	0.45 M in Methanol	99 after 900 s	90	$Pd_{25}Zn_{75}/TiO_2$	333 K, 5 bar H_2	Wall-coated microreactor	5
Thermal	0.011 M in Methanol	96 after 36 h	81	$Pd_{25}Zn_{75}/TiO_2$	333 K, 5 bar H_2	Batch	5
Themal	0.1 M MBY in Methanol	95	81	Polymer-protected PdZn	333 K, 5 bar H_2	Batch	6
Thermal	0.9 M in Ethanol	95 after 30 min	83	Pd supported on hypercrosslinked polysterene	333 K, 3 bar H_2	Batch	7
Thermal	0.25 M in EtOH	20 after 120 min	90	Lindlar Catalyst	25°C, 800 rpm, 1 bar H_2	Batch	8
Thermal	Gas-phase	70	80	Pd/ZnO	403 K Atmospheric pressure	Gas-phase Continuous flow	9
Thermal	Gas-phase	80	60	Pd/Al_2O_3	403 K Atmospheric pressure	Gas-phase Continuous flow	9
Thermal	Gas-phase	80	20	Lindlar	403 K Atmospheric pressure	Gas-phase Continuous flow	9
Thermal	0.67 M MBY in Toluene	96 after 8	39	Functionalized Ni-NPs	Room Temperature, 1 bar H_2	Batch	10
Electro	0.49 M MBY in EtOH/KOH	Ca. 100 % after 256 h	42	PdFe NSs	-0.4 V vs. RHE	Batch	11
**Electro**	**1 M MBY in 0.3 M KOH /H_2O**	**60 after 2 h**	**71**	**$Fe_3Ni_6S_8$**	**80 mA cm^{-2} Ambient conditions**	**Semi continuous**	**This work**
**Electro**	**1 M MBY in 0.3 M KOH /H_2O**	**80 after 2 h**	**29**	**$Fe_3Ni_6S_8$**	**240 mA cm^{-2} Ambient Conditions**	**Semi continuous**	**This work**

Hydrogenation of MBY: Conversion factors & Reaction rates

The reaction rate r (in h^{-1}) was calculated according to Eq. 4:

$$r = C_{MBY} \frac{X}{100} \cdot \frac{V}{t \cdot n_{Fe3Ni6S8}}, \tag{4}$$

where C_{MBY} is the concentration of MBY at the start of reaction, X the conversion, V is the reservoit volume, while t and $n_{Fe3Ni6S8}$ correspond to the total time of electrolysis and moles of catalyst used.

At catalytic loading of 5 mg cm^{-2} was used, amounting to 62.85 mg of catalyst. For the used $Fe_3Ni_6S_8$ catalyst this corresponds to $8.108 \cdot 10^{-5}$ mol, with the time of electrolysis being 2 h.

Table S4. Calculation of the achieved conversion and reaction rate depending on the applied current density.

	j / mA cm^{-2}			
	40	80	160	240
n(MBY) in catholyte / mol	0.0366	0.0253	0.0181	0.0151
Conversion / %	26.8	49.4	63.8	69.8
Reaction rate / 10^2 h^{-1}	0.86	1.5	1.9	2.1

Furthermore, we calculated the activity (A) of the catalyst in mol $mol_{catalyst}$ s^{-1} according to the following equation (**Eq. 5**) and compared it to the activity values of the report by Johnston *et al.*[12]:

$$A = \frac{(n_{MBY,initial} - n_{MBY,end})}{t \cdot n_{Fe3Ni6S8}} \tag{5}$$

in which $n_{MBY,Initial}$ is the amount of MBY at the start of the reaction (0.05 mol) and $n_{MBY,End}$ the detected amount of MBY in the catholyte at the end of the electrolytic reaction. With t being total time of electrolysis in seconds, and $n_{Fe3Ni6S8}$ and moles of used catalyst.

Table S5. Calculation of the achieved activity depending on the applied current density.

	j / mA cm^{-2}			
	40	80	160	240
Activity / mol mol$^{-1}_{Fe3Ni6S8}$ s^{-1}	0.02	0.04	0.05	0.06

Table S6 Comparison of the herein obtained activity data from the ECH of MBY employing pentlandite electrodes against the thermocatalytic state-of-the-art.[12]

Catalyst - Process	Activity mol mol^{-1}catalyst s^{-1}
$Fe_3Ni_6S_8$ – Electrochemical	0.06
Pd/TiO_2	0.42
Pd_3Sn/TiO_2	0.22
Pd/ZnO	0.88
Pd_3Sn/ZnO	0.06

Long-term ECH experiments with pentlandite-based electrodes

To assess the catalytic stability of our Ni_6 catalyst, the ECH was performed for 100h at 160 mA cm^{-2} with the optimal cathode parameters. The electrolyte volume was set to 938 L to enable a theoretical conversion to MBE of 100% in 25h and ensure necessary laboratory safety precautions. The catholyte and anolyte were exchanged with fresh solutions after every 25h until the 100h mark.

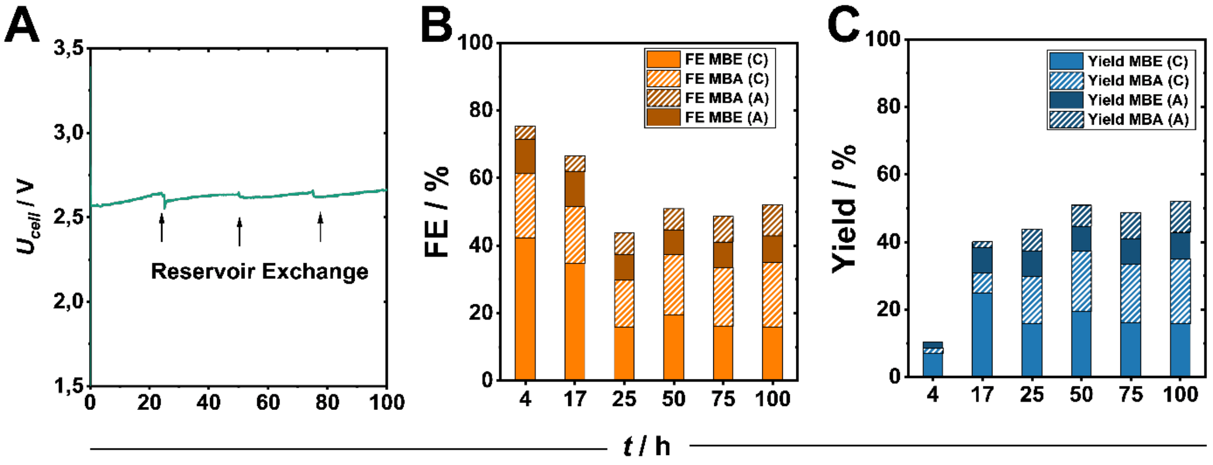

Figure S17. Investigation of the herein employed pentlandite-electrode and electrolyzer at 160 mA cm^{-2} in 0.3 M KOH for 100 h of electrolysis, at a flow rate of 12 ml ml^{-1} in batch-mode. The obtained cell voltage (A), faradaic efficiency (B) and yield (C) through ^{1}H-NMR quantification. Here FE_{MBX}(C) and FE_{MBX}(A) (X: E, A) correspond to the FE values of ECH products detected in the catholyte and anolyte after electrolysis, respectively.

References

1 D. Tetzlaff, K. Pellumbi, D. M. Baier, L. Hoof, H. Shastry Barkur, M. Smialkowski, H. M. A. Amin, S. Grätz, D. Siegmund, L. Borchardt and U.-P. Apfel, *Chemical science*, 2020, **11**, 12835.

2 K. Pellumbi, M. Smialkowski, D. Siegmund and U.-P. Apfel, *Chemistry (Weinheim an der Bergstrasse, Germany)*, 2020, **26**, 9938.

3 S. Vernuccio, R. Goy, P. Rudolf von Rohr, J. Medlock and W. Bonrath, *React. Chem. Eng.*, 2016, **1**, 445.

4 M. Crespo-Quesada, M. Grasemann, N. Semagina, A. Renken and L. Kiwi-Minsker, *Catalysis Today*, 2009, **147**, 247.

5 E. V. Rebrov, E. A. Klinger, A. Berenguer-Murcia, E. M. Sulman and J. C. Schouten, *Org. Process Res. Dev.*, 2009, **13**, 991.

6 L. B. Okhlopkova, E. V. Matus, I. P. Prosvirin, M. A. Kerzhentsev and Z. R. Ismagilov, *J Nanopart Res*, 2015, **17**, 1.

7 L. Nikoshvili, E. Shimanskaya, A. Bykov, I. Yuranov, L. Kiwi-Minsker and E. Sulman, *Catalysis Today*, 2015, **241**, 179.

8 Y. Yang, X. Zhu, L. Wang, J. Lang, G. Yao, T. Qin, Z. Ren, L. Chen, X. Liu, W. Li and Y. Wan, *Nat Commun*, 2022, **13**, 2754.

9 A. González-Fernández, C. Pischetola, L. Kiwi-Minsker and F. Cárdenas-Lizana, *J. Phys. Chem. C*, 2020, **124**, 3681.

10 A. M. López-Vinasco, L. M. Martínez-Prieto, J. M. Asensio, P. Lecante, B. Chaudret, J. Cámpora and P. W. N. M. van Leeuwen, *Catal. Sci. Technol.*, 2020, **10**, 342.

11 K. Zhu, J. Ma, L. Chen, F. Wu, X. Xu, M. Xu, W. Ye, Y. Wang, P. Gao and Y. Xiong, *ACS Catal.*, 2022, **12**, 4840.

12 S. K. Johnston, N. Cherkasov, E. Pérez-Barrado, A. Aho, D. Y. Murzin, A. O. Ibhadon and M. G. Francesconi, *Applied Catalysis A: General*, 2017, **544**, 40.
